# The toxicity of cadmium and resulting hazards for human health

**DOI:** 10.1186/1745-6673-1-22

**Published:** 2006-09-10

**Authors:** Johannes Godt, Franziska Scheidig, Christian Grosse-Siestrup, Vera Esche, Paul Brandenburg, Andrea Reich, David A Groneberg

**Affiliations:** 1 Department for Paediatric Pneumology and Immunology, Charité – School of Medicine, Free University and Humboldt University of Berlin, Germany; 2 Institute of Occupational Medicine, Charité – School of Medicine, Free University and Humboldt University of Berlin, Germany; 3 Department of Comparative Medicine and Experimental Animal Sciences, Charité – School of Medicine, Free University and Humboldt University of Berlin, Germany

## Abstract

Cadmium (Cd) has been in industrial use for a long period of time. Its serious toxicity moved into scientific focus during the middle of the last century. In this review, we discuss historic and recent developments of toxicological and epidemiological questions, including exposition sources, resorption pathways and organ damage processes.

## Background

Cadmium (group IIB of the periodic table of elements) is a heavy metal posing severe risks to human health. Up to this day, it could not be shown that cadmium has any physiological function within the human body. Interest has therefore risen in its biohazardous potential. As first described by Friedrich Stromeyer (Göttingen, Germany) in 1817, cadmium intoxication can lead to kidney, bone, and pulmonary damages.

In this article, we review recent developments and findings of cadmium toxicology.

## Occurrence

Cadmium is regularly found in ores together with zinc, copper and lead. Therefore volcanic activity is one natural reason for a temporary increase in environmental cadmium concentrations. Cadmium is widely used in industrial processes, e.g.: as an anticorrosive agent, as a stabilizer in PVC products, as a colour pigment, a neutron-absorber in nuclear power plants, and in the fabrication of nickel-cadmium batteries. Phosphate fertilizers also show a big cadmium load. Although some cadmium-containing products can be recycled, a large share of the general cadmium pollution is caused by dumping and incinerating cadmium-polluted waste [1]. In Scandinavia for example, cadmium concentration in agricultural soil increases by 0.2% per year. Total global emission of cadmium amounts to 7000 t/year [2].

## Resorption into human body

The maximum permissible value for workers according to German law is 15 μg/l. For comparison: Non-smokers show an average cadmium blood concentration of 0.5 μg/l.

Basically there are three possible ways of cadmium resorption: Gastrointestinal, pulmonary and dermal.

### Digestive system

The uptake through the human gastrointestinal is approximately 5% of an ingested amount of cadmium, depending on the exact dose and nutritional composition [3]. An average German citizen has a daily intake of 30–35 μg cadmium; 95% of this taken up with food and drinks. An average smoker has an additional intake of 30 μg per day [4]. Several factors can increase this amount, such as low intakes of vitamin D, calcium, and trace elements like zinc and copper.

Concerning zinc and calcium, it is assumed that their molecular homology could be a reason for a compensatory higher cadmium resorption [5]. Foulkes was able to show such a competitive resorption of Cd in an animal model: In rat jejunum, the cadmium uptake was depressed by relatively high concentrations of other polyvalent cations, including Pb, Ni, Cr3+, Sr, and Mg [6].

Furthermore a high fiber diet increases the dietary cadmium intake [7]. The most important metabolic parameter for cadmium uptake is a person's possible lack of iron. People with low iron supplies showed a 6% higher uptake of cadmium than those with a balanced iron stock [8]. This is the main reason for the higher cadmium resorption in people with anaemia and habitual iron deficit, such as children or menstruating women. Low iron blood levels stimulate the expression of DCT-1, a metal ion transporter in the GI tract, serving as a gate for cadmium resorption [9].

### Respiratory system

The major source of inhalative cadmium intoxication is cigarette smoke. The human lung resorbes 40–60% of the cadmium in tobacco smoke [10]. A 50 year-old average non-smoker has a cadmium body burden of 15 mg. While a comparable life-long smoker shows a value of 30 mg. Smokers generally have cadmium blood levels 4–5 times those of non-smokers [7].

Workers exposed to cadmium-containing fumes have been reported to develop acute respiratory distress syndromes (ARDS) [11].

Inhalativly resorbed cadmium reaches blood circulation usually in form of cadmium-cysteine complexes [12].

### Dermal resorption

Little research has been done on dermal absorption of cadmium. In 1991, Wester et al. experimented on the resorption from cadmium-contaminated soil and water solutions by human cadaver skin in a diffusion cell-model. They could demonstrate a penetration of 8.8 % (soil) and 12.7% (water) of the applied cadmium dose into the skin; while the plasma uptake from soil was 0.01% and 0.07% from water [13]. Lansdown and Sampson administered a cadmium chloride solution to the dorsum of rats (shaved skin) daily for 10 days. The skin showed hyperkeratosis and acanthosis with occasional ulcerative change, and an increase of the mitotic index of the skin cells. Also cadmium concentration in blood, liver and kidney increased, thus indicating percutaneous absorption [14].

Two mechanisms facilitate cadmium absorption by the skin: binding of a free cadmium ion to sulfhydryl radicals of cysteine in epidermal keratins, or an induction and complexing with metallothionein [15].

### Handling Of cadmium in the body

Once taken up by the blood, the majority of cadmium is transported bound to proteins, such as Albumin and Metallothionein.

The first organ reached after uptake into the GI-blood is the liver. Here cadmium induces the production of Metallothionein. After consecutive hepatocyte necrosis and apoptosis, Cd-Metallothionein complexes are washed into sinusoidal blood. From here, parts of the absorbed cadmium enter the entero-hepatical cycle via secretion into the biliary tract in form of Cadmium-Glutathione conjugates. Enzymatically degraded to cadmium-cysteine complexes in the biliary tree, cadmium re-enters the small intestines [12].

The main organ for long-term cadmium accumulation is the kidney [16]. Here the half-life period for cadmium is approx. 10 years. A life-long intake can therefore lead to a cadmium accumulation in the kidney, consequently resulting in tubulus cell necrosis.

**Figure 1:**
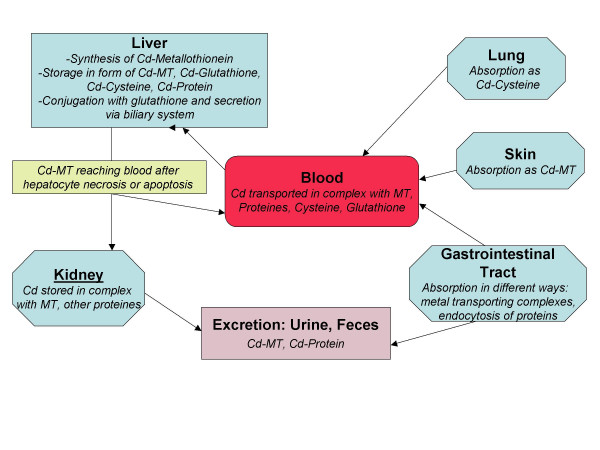
**Handling of cadmium in human body**. Figure legend text: Metabolism, storage and excretion of cadmium in human body. Modified after [12].

The blood concentration of cadmium serves as a reliable indicator for a recent exposition, while the urinary concentration reflects past exposure, body burden and renal accumulation [3]. Excretion of Cadmium takes place via faeces and urine. Figure 1 gives a scheme on the handling of Cadmium in human body.

## Hazards to human health

### Acute intoxication

The respiratory system is affected severely by the inhalation of cadmium-contaminated air: Shortness of breath, lung edema and destruction of mucous membranes as part of cadmium-induced pneumonitis are described [17]. As already reported in 1942, intake of cadmium-contaminated food causes acute gastrointestinal effects, such as vomiting and diarrhoea [18].

### Kidney damage

Kidney damage has long since been described to be the main problem for patients chronically exposed to cadmium [19]. As mentioned above, cadmium reaches the kidney in form of cadmium-metallothionein (Cd-MT). Cd-MT is filtrated in the glomerulus, and subsequently reabsorbed in the proximal tubulus. It then remains in the tubulus cells and makes up for the major part of the cadmium body burden. The amount of cadmium in the kidney tubulus cells increases during every person's life span. A perturbance of the phosphor and calcium metabolism as a result of this phenomenon is in discussion [20]. An increasing cadmium load in the kidney is also discussed to result in a higher calcium excretion, thus leading to a higher risk of kidney stones.

The urinary cadmium excretion was shown to correlate with the degree of cadmium induced kidney damage: A urinary excretion of 2.5 micrograms cadmium per gram creatinine reflects a renal tubular damage degree of 4% [7]. The primary markers of kidney damage however, are the urinarily excreted β2-microglobulin, N-acetyl-α-D-glucosaminidase (NAG), and retinol-binding-protein (RBP) [21]. The ChinaCad-Study showed significantly higher values for urinary β2-Microglobulin and RBP in people with high blood cadmium concentration than in people with normal values [3]. In the first group, both glomerular and tubular damages where observed. It has been discussed whether or not tubular damage is reversible [22]. The general opinion today however is, that it's irreversible.

### Effects of cadmium in reproductive biology

Cadmium appears to interfere with the ovarian steroidogenic pathway in rats. Piasek et al. evaluated the direct effects of in vitro cadmium exposure on steroidogenesis in rat ovaries.

The most affected were productions of progesterone and testosterone [23]. Low dosages of cadmium are reported to stimulate ovarian progesterone biosynthesis, while high dosages inhibit it [24]. Maternal exposure to cadmium is associated with low birth wight and an increase of spontaneous abortion [25,26]. Some evidence exists also that cadmium is a potent nonsteroidal estrogen in vivo and in vitro. Studies in rats showed that cadmium precipitates enhanced mammary development and increased uterine wight [27].

### Bone damage and the Itai-Itai-disease

Several studies in the 20^th ^Century showed a connection between cadmium intoxication and bone damage, e.g. in workers exposed to cadmium-polluted fume and dust [28].

Cadmium could also be shown to be associated with occurrences of Itai-Itai, a disease under witch patients show a wide range of symptoms such as: low grade of bone mineralization, high rate of fractures, increased rate of osteoporosis, and intense bone associated pain. An epidemic occurrence of the Itai-Itai disease was observed in the Jinzu river basin (Japan) in the 1940s. In a study on this occasion, patients where found to show the characteristic symptoms after having eaten rice, grown on fields irrigated with highly cadmium polluted water. Also pseudo fractures characteristic of osteomalacia and severe skeletal decalcification could be observed. Criticism of this study came up because of the fact that the majority of the patient collective was made up of women in the post-menopause [29]. Underlying osteoporosis, possibly enhanced by cadmium intoxication, was suggested to be the actual reason for the observed symptoms [30].

Further evidence for the causality of cadmium intoxication for bone maladies was found in 2003 by Honda et al. They could describe an inverse correlation of the STIFF index (an ultrasound method for measuring bone density) and urine cadmium concentration [25]. Similar findings where made within the OSCAR-Study, conducted with 1021 people from southern Sweden. Here a significant negative correlation could be shown between urine cadmium concentration and low bone mineral density; especially in people of an age of 60 years and above. Furthermore evidence for an increased risk of forearm fractures in cadmium-exposed individuals was found [31]. Individuals included in this study were either battery plant workers, or inhabitants of a town close to the battery plant. A collective of unexposed people where included as reference group.

The Belgian CadmiBel study – conducted between 1985 and 1989 – came to similar conclusions: Even minimal environmental exposure to cadmium is supposed to cause skeletal demineralisation [32]. Some of the CadmiBel-participants were later tested for forearm bone density during the so called PheeCad Study (1992–1995). Here too lower bone densities where found in individuals previously exposed to cadmium. The most interesting aspect of this study was the fact, that their total cadmium body burden (according to the urinary cadmium excretion) was significantly lower than that of Japanese Itai-Itai patients: CadmiBel/PheeCad participants showed a urinary cadmium excretion of only 1 μg/g creatinine, while Itai-Itai patients where found to have an excretion of approximately 30 μg/g creatinine.

**Figure 2:**
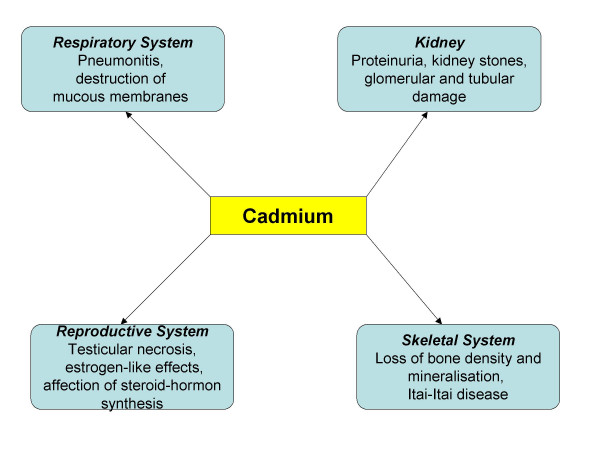
Effects of cadmium on several organ systems.

The exact mechanism of interference between cadmium and bone mineralization remains to be discovered. Presently, a direct influence on osteoblast and osteoclast function seems as likely as an indirect influence via induction of renal dysfunction [33]. A perturbance of the vitamin D3 metabolic pathway through cadmium is also in discussion: According to these hypothesises, lead and cadmium interact with renal mitochondrial hydroxylases of the vitamin D3 endocrine complex [34]. Figure 2 gives an overview on the effects of cadmium in several organ systems.

### Carcinogenity

There is some proof that cadmium can cause cancer. Waalkes et al. have shown that a subcutaneous injection of cadmium chloride can induce prostate cancer in Wistar rats [35]. This group also postulated that high doses of cadmium can cause severe testicular necrosis in rats, followed by a higher incidence of testicular interstitial tumors. In contrast to laboratory data though, epidemiological studies could not convincingly prove cadmium to be a cause of prostate cancer [36].

Early publications however suggested an association of cadmium and renal cancer in humans [37]. This assumption was confirmed in 2005 by a systematic review of seven epidemiological and eleven clinical studies [38]. Consequently, the IARC (International Agency for Research on Cancer) decided to classify cadmium as a human carcinogen group I. Latest data however supports the assumption that only an uptake of cadmium via the respiratory system has carcinogenic potential [3].

Although molecular mechanisms of cadmium-induced carcinogenesis are not yet understood, several factors may contribute to it: Up-regulation of mitogenic signalling, perturbance of DNA-repairing mechanism, and acquisition of apoptotic resistance by cadmium exposure [39]. A substitution of zinc by cadmium in transcription-regulating proteins is also in discussion. Furthermore, new data showed that cadmium is able to change the conformation of E-Cadherin, a transmembrane Ca(II)-binding glycoprotein. E-Cadherin plays an important role in cell-cell adhesions, especially in epidermal cells [40]. These results are consistent with the hypothesis that E-cadherin may be a direct molecular target for Cd(2+) toxicity.

There are many further fields of occupational medicine and toxicology in which cadmium is currently suspected to play a major role [41-45] They are omitted with regard to the limited space and the comprehensiveness of this review.

## Conclusion

Latest studies have proven the importance of a reduction of cadmium emissions for human health. Some efforts in this direction have been made, especially within in the European Union. Cadmium, on the one hand, is example for an industrially used substance with negative long-time effects on human health. On the other hand, it is an example for the beneficial potential of the international cooperation of laboratories, universities and local authorities. Efforts to research and reduce the effects of cadmium emissions have to continue. A number of promising projects give rise to the hope that, in the future, alternative testing methods may allow a reduction of the number of laboratory animals necessary for this research.

**Table 1:** Recent studys on Cadmium toxicity

Study, year of publication	Localisation	Total number of participants	Main points of interest
ChinaCad, 2002	Wenzhou City area, China	790	Cadmium biomonitoring, renal dysfunction
OSCAR, 2004	Fliseryd area, Sweden	1021	Renal and bone effects of low-level cadmium exposure
CadmiBel, 1985–1990 Follow-up by PheeCad-study	Liege, Charleroi and rural areas	2327	Several cadmium effects on human body
